# Predicting nursing home admission in the U.S: a meta-analysis

**DOI:** 10.1186/1471-2318-7-13

**Published:** 2007-06-19

**Authors:** Joseph E Gaugler, Sue Duval, Keith A Anderson, Robert L Kane

**Affiliations:** 1 Center on Aging, Center for Gerontological Nursing, School of Nursing, University of Minnesota, 6-150 Weaver-Densford Hall, 1331, 308 Harvard Street S.E., Minneapolis, MN, 55455 USA; 2 Division of Epidemiology and Community Health, School of Public Health, University of Minnesota, Division of Epidemiology & Community Health, 1300 South Second Street, Suite 300, Minneapolis, MN 55454-1015 USA; 3 Graduate Center for Gerontology, College of Public Health, University of Kentucky, 304H Charles T Wethington Building 0200, Lexington, KY 40506 USA; 4 Division of Health Policy and Management, University of Minnesota School of Public Health, Mayo Mail Code 197, 420 Delaware Street SE, Minneapolis, MN 55455 USA

## Abstract

**Background:**

While existing reviews have identified significant predictors of nursing home admission, this meta-analysis attempted to provide more integrated empirical findings to identify predictors. The present study aimed to generate pooled empirical associations for sociodemographic, functional, cognitive, service use, and informal support indicators that predict nursing home admission among older adults in the U.S.

**Methods:**

Studies published in English were retrieved by searching the MEDLINE, PSYCINFO, CINAHL, and Digital Dissertations databases using the keywords: "*nursing home placement*," "*nursing home entry*," "*nursing home admission*," and "*predictors/institutionalization*." Any reports including these key words were retrieved. Bibliographies of retrieved articles were also searched. Selected studies included sampling frames that were nationally- or regionally-representative of the U.S. older population.

**Results:**

Of 736 relevant reports identified, 77 reports across 12 data sources were included that used longitudinal designs and community-based samples. Information on number of nursing home admissions, length of follow-up, sample characteristics, analysis type, statistical adjustment, and potential risk factors were extracted with standardized protocols. Random effects models were used to separately pool the logistic and Cox regression model results from the individual data sources. Among the strongest predictors of nursing home admission were 3 or more activities of daily living dependencies (summary odds ratio [OR] = 3.25; 95% confidence interval [CI], 2.56–4.09), cognitive impairment (OR = 2.54; CI, 1.44–4.51), and prior nursing home use (OR = 3.47; CI, 1.89–6.37).

**Conclusion:**

The pooled associations provided detailed empirical information as to which variables emerged as the strongest predictors of NH admission (e.g., 3 or more ADL dependencies, cognitive impairment, prior NH use). These results could be utilized as weights in the construction and validation of prognostic tools to estimate risk for NH entry over a multi-year period.

## Background

The cost of nursing home (NH) care for persons 65 years of age and over is estimated to be roughly 150 billion dollars by 2007 in the United States (U.S.). About 62% of this cost in the U.S. is assumed by public, taxpayer-financed sources such as Medicaid and Medicare [1]. Not only is admission to NHs expensive, it is associated with a number of other problematic outcomes such as questionable quality of care, early mortality for many residents, and psychological or emotional upheaval for caregiving families [2-4]. The constellation of potentially negative outcomes associated with NH admission for older adults has encouraged a search for ways to divert or delay individuals from entering long-term care facilities. Over the past 25 years, many observational studies have attempted to identify predictors of NH entry with the goal of improving preadmission assessment of older adults.

Several comprehensive literature reviews have summarized studies predicting NH admission among older adults [5-9]. These reviews address "long-term" predictors of NH admission, or those factors that influence NH entry 1 year or more in the future. Variables found to consistently predict NH admission include sociodemographic and background factors such as increased age, Caucasian ethnicity/race, living alone, and female gender. Other variables reflect the functional independence of older adults, such as greater activity of daily living (ADL) dependence or cognitive impairment. Additional predictors of NH admission move beyond the older adult to capture the care received (e.g., unavailable family caregiver; community-based service use) or community contexts (lower NH bed supply).

While there is some consensus regarding the identification of singular factors that predict NH admission for older adults 1 year or more into the future, there are several limitations in existing research. One is the reliance on samples that are not representative of the older U.S. population. This weakness is apparent not only in individual studies, but also systematic reviews of predictors of NH entry as attempts to integrate existing findings often include non-representative data sources along with representative ones [5-7]. This may influence the generalizability of the conclusions, which often seek to provide a synthesis of those factors that predict NH entry for older adults in the U.S. Existing reviews also fail to account for multiple studies using the same databases. Many studies are often conducted on a handful of data sources such as the Longitudinal Study on Aging (LSOA) [10-12] or the National Long-Term Care Survey (NLTCS) [13-15]. Such a procedure may skew the combined estimates, as those data sources with greater individual study representation would have an inordinate influence on conclusions of prediction.

Most studies of NH admission focus on the factors that antedate entry by several years and serve as early warning signs of those at greatest risk for entry. This study systematically reviews the long-term predictors of NH admission (i.e., predictors of NH entry 1 year or more in the future) for U.S. older adults in the community. Deriving estimates of prediction based on generalizable data sources may inform the development of useful prognostic tools to help identify who is at-risk for NH entry at some time point in the future. For example, the generation of pooled empirical associations via a meta-analysis of this type could serve as empirical weights in the development of predictive algorithms of NH admission for community-residing older adults [16].

The findings of this meta-analysis can confirm significant predictors of NH admission identified in prior, well-executed systematic reviews [5-7]. However, using a meta-analysis offers several advantages to these excellent reviews by permitting the generation of empirical estimates of effect strength for each predictor of NH admission. This meta-analysis builds on the findings of past reviews, which summarize the direction of associations or make qualitative interpretations of the strength of associations between predictor variables and NH entry. Moreover, as alluded to above, synthesizing the empirical associations of various predictors across nationally- and regionally-representative data sources via a meta-analysis (as opposed to combining representative data sources with non-representative ones) provides greater external validity for pooled results and yields more generalizable information on those factors that predict NH admission. For these reasons, the objective of this meta-analysis was to extend the work of prior reviews to offer more definitive findings on those variables that emerge as significant predictors of NH admission for community-residing older adults.

## Methods

### Search of the literature and study inclusion

Published analyses were initially located through a search of the MEDLINE, CINAHL, PSYCINFO, and Dissertation Abstracts/Digital Dissertation databases using the following key terms: "*nursing home placement*," "*nursing home entry*," "*nursing home admission*," and "*predictors/institutionalization*." Any reports including these key words were retrieved. The principal author, who has helmed several studies on NH admission in dementia [17,18], screened study abstracts for inclusion and extracted all data (see below). Dates of publication were limited to 1950-present, and the comprehensive database search occurred in February/June, 2005 and again in March, 2006. Exclusion criteria included studies conducted in a non-community setting (e.g., hospital discharge analyses) and evaluations of a pharmacological or non-pharmacological intervention. Following the database search, the principal author engaged in an intensive cross-referencing procedure of each selected report as well as prior literature reviews; 13 iterations were completed until no unique references were identified. In instances where a report could not be retrieved, the principal author sent two email queries to corresponding authors.

### Data extraction

A standardized extraction protocol was used. Inclusion criteria were as follows: 1) NH entry was an outcome in the analysis; 2) the study was conducted within the U.S. due to the diverging care philosophies, regulatory environment, and service delivery approaches of NH care in other countries; 3) the study design was longitudinal in order to allow for a predictive analysis of NH admission; 4) the sample was general, as opposed to disease-specific (e.g., dementia); 5) the sample resided in a community setting, such as at home alone or with relatives, as opposed to a healthcare setting (e.g., hospital discharge studies); and 6) the sample was regionally- or nationally-representative of the U.S. population of older adults in order to maximize the generalizability of the pooled estimates. The screening protocol also extracted date of publication, source of publication, author, sample size, percentage of older adults (65 years of age and over) and women in each sample, and type of data collected (e.g., survey, clinical rating, medical charts/records, etc.). For eligible studies the principal author extracted data for each reported predictor of NH admission, including regression coefficients, standard errors, odds/hazard ratios, and lower/upper confidence intervals. Data on study design included analysis type, length of follow-up, classification of long- or short-stay NH admissions, and analytic unit of predictors (e.g., individual-level, community-level, state-level, etc.).

### Statistical analysis

Results were integrated with random effects models, as these models consider both within- and between-study variation when deriving pooled empirical associations [17]. Heterogeneity was assessed by Cochran's Q-statistic, with a value of *P *< .05 indicating that study results were heterogeneous [19]. Sets of studies that are classified as heterogeneous may not assess the same empirical relationships between an independent variable of interest (i.e., exposure) and the outcome of interest; thus, differences in study results may be a result of factors other than random variation. Meta-regression models were considered to investigate heterogeneity of empirical associations (e.g., age of study, size of study), but the number of data sources for each predictor variable precluded such analyses.

A detailed variable cross-walk identified individual predictors of NH admission and their operational meaning across individual studies. This allowed for a much clearer interpretation of results in the meta-analysis, as pooled estimates for each predictor were based on variables that were closely operationalized or measured across studies as opposed to variables that were grouped in more general categories (e.g., "functional status"). Almost all the studies included only reported adjusted estimates; therefore, these estimates were used for pooling purposes. Results from logistic regression models (odds ratios) and Cox regression/survival models (hazard ratios) were analyzed separately, as these two approaches address related but different questions. Logistic regression models explore whether NH admission occurred or not, whereas Cox regression models examine the time to NH admission. Meta-analysis procedures were conducted separately for each analysis type to further clarify the meaning of pooled empirical associations [20].

In a few instances, multiple studies from the same data source provided empirical information on a predictor of interest. When this occurred, the principal author compared the competing studies to identify the presence of seven important study characteristics: 1) more comprehensive adjustment used in the analysis (i.e., number of predictors of NH admission included); 2) greater sample size; 3) longer follow-up; 4) use of clinical rating of functional or cognitive data; 5) use of objective data on NH admission (e.g., death certificate); 6) categorization of admission included (i.e., long-stay vs. short-stay); and 7) use of multiple levels of predictors (individual-level; community-level). The study that included a greater number of these criteria was selected for the meta-analysis.

## Results

### Identification of studies

**Figure 1:**
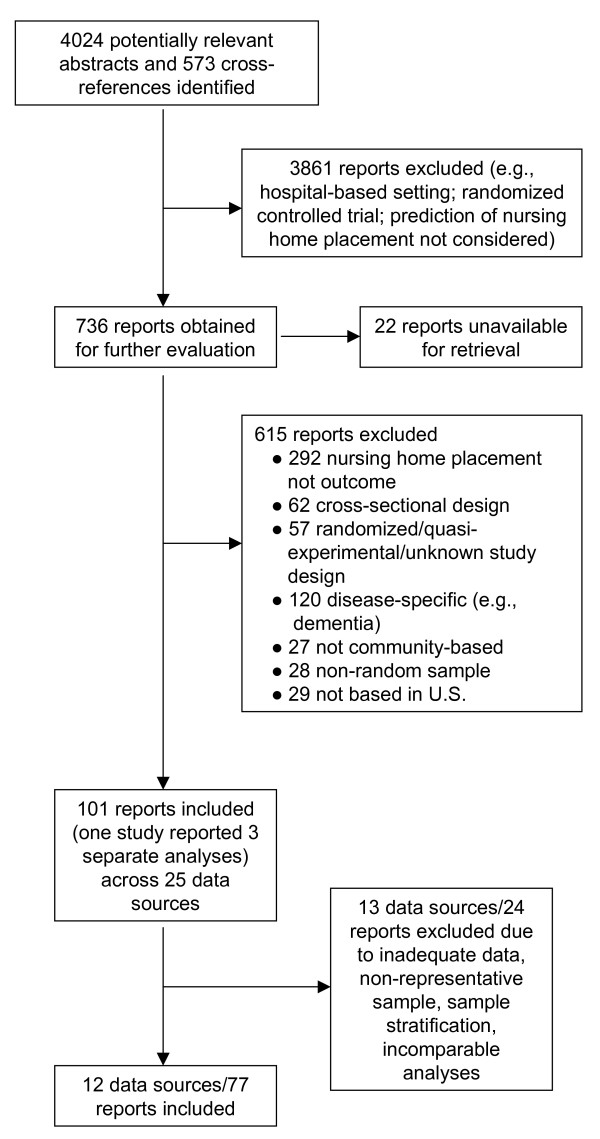
Identification of eligible studies and data sources.

Of 4,597 abstracts initially screened, 3,861 were excluded because these studies did not consider predictors of NH admission, were evaluations of pharmacological or non-pharmacological interventions, or took place in settings other than the community (e.g., hospital; see Figure 1). Of the 736 reports retrieved for initial screening procedures, 22 were not available following two email contacts with the authors or use of interlibrary loan services. A further 615 reports were excluded from the meta-analysis based on failing the inclusion criteria specified above, leaving 99 studies. One study [21] presented results within three different data sources, and each of these analyses were treated as a separate "study" resulting in the identification of 101 studies reporting from 25 various data sources.

Of these 25 original data sources, 12 were found to have studies that yielded data appropriate for the current meta-analysis. The remaining data sources were not included for a range of reasons, such as incomplete data, race/gender specific analyses, the data source was not representative of a region or the entire U.S. elderly population, or the outcome analyzed was not comparable to those of the logistic regression and Cox regression models and could not be pooled (e.g., multinomial logistic regression or probit models). A final total of 77 reports were considered across these 12 data sources.

### Data source characteristics

Pooled together at their capacity, the 12 data sources represent 178,056 older adults (65 years of age and over; see Table 1). Five of the data sources included nationally representative sampling frames, whereas the other 7 were random samples from specific geographic locations of the U.S. Two data sources included older adults with some level of functional disability: the National Long-Term Care Survey [13-15,22-25] and the Massachusetts Elder Health Care Project [26]. Two data sources limited their national samples to non-institutionalized adults 70 years of age and over: the Longitudinal Study of the Aged [10-12,27] and the Study of Assets and Health Dynamics of the Oldest Old [28-30]. The sample sizes available in each data source varied widely from 586 (Massachusetts Elder Health Care Project) [26] to 137,632 (Medicare Health Outcomes Study) [31]. Data collection was largely via interviews, either directly with the elderly respondent or a proxy. Length of follow-up/exposure also varied from 1 to 10 years [32,33]. Although specific measures of each dimension varied, most data sources included comparable types of predictor variables such as sociodemographic characteristics, functional dependency, cognitive impairment, and to a lesser extent formal service utilization and informal/family support.

**Table 1:** Characteristics of 12 Data Sources Included in Meta-Analysis of Predictors of Nursing Home Admission.

**Data Source**	**Sampling Frame**	**Sample Size**	**Data Collection**	**Duration of Follow-Up**	**Number of Nursing Home Admissions/Rate**	**Predictor Variables Available**
Study of Assets and Health Dynamics of the Oldest Old (AHEAD)	National panel study, 70 years of age and over in U.S.	Up to 8,222	Baseline face-to-face interviews, telephone follow-ups every other year with respondent or proxy	Up to 7 years (1993–2000)	Up to 1135/	Cognitive performance, physical and functional health, economic status, family structure, demographics, service use, caregiving, financial resources
Established Populations for Epidemiological Studies of the Elderly-East Boston (EPESE-EB)	All non-institutionalized persons 65 years age and over in East Boston, Massachusetts enumerated via community census	3,545	In-person and telephone interview with respondent and/or proxy	1982–1985	142	Demographic characteristics, social and physical functioning, chronic Conditions, related health problems, self-reported Service use, depression
Established Populations for Epidemiological Studies of the Elderly-Iowa (EPESE-IOWA)	All non-institutionalized persons 65 years age and over in Washington and Iowa counties, enumerated via all Area Agencies on Aging and local informants	Up to 3,097	"	Up to 4 years (1982–1986)	Up to 369	"
Established Populations for Epidemiological Studies of the Elderly-New Haven (EPESE-NH)	All non-institutionalized persons 65 years age and over in New Haven, Connecticut enumerated via stratified random sample of household clusters	Up to 2,812	"	Up to 9 years (1982–1991)	Up to 935	"
Government Accounting Organization-Cleveland Study (GAO)	Older adults, 65 years and age and over and living independently, were randomly selected in Cleveland, OH	1,598	In-person interviews with respondent or proxy	1975–1984	406	Sociodemographics, mental health, physical health, disability, available informal support
Longitudinal Study on Aging (LSOA)	All civilian non-institutionalized persons in the U.S. 70 years of age and over in 1984	Up to 7,541	In-person interviews at baseline, telephone interviews at each 2-year follow-up with respondent or proxy	Up to 6 years (1984–1990)	Up to 1100	Cognitive performance, physical and functional health, economic status, family structure, demographics, service use, caregiving, financial resources
National Long-Term Care Survey (NLTCS)	Random sample from Medicare enrollment files, 65 years of age and over in U.S. with any ADL or IADL dependency	Up to 5,851	In-person interviews with older respondent and/or proxy	1982–1984	Up to 911	Cognitive performance, physical and functional health, economic status, family structure, demographics, service use, caregiving, financial resources
Medicare Survey	People living in the community during the 1977 Current Medicare Survey	4400	Interviews with respondents initially and one year later	1977–1978	127	Sociodemographics, payment source, functional status, perceived health status, service use and physician contact
Massachusetts Elder Health Project	A geographically stratified random sample of disabled elders living in Eastern Massachusetts	Up to 586	In-person and telephone interviews with respondent or proxy	Up to 6 years (1984/85–1991)	Up to 143	Informal and formal community care, caregiving burden, sociodemographics, physical disability
Massachusetts Health Care Panel Study (MHCPS)	Statewide (Massachusetts) probability sample of non-institutionalized adults 65 years of age and over	1625	In-person interviews at baseline and first follow-up a year later; mailed surveys every 4 years thereafter	Up to 10 years (1974/75–1984/85)	Up to 148	Demographics, social characteristics, functional disabilities, attitudinal variables
Medicare Health Outcomes Study (MHOS)	Random sample of Medicare+Choice (Medicare managed care) enrollees over the age of 64 who were not institutionalized	137,632	Mail survey and telephone follow-up of non-respondents on an annual basis	1998–2000	11,220	Quality of life/psychosocial status, self-reported symptoms and diagnoses, functional disability, sociodemographics
Monogohela Valley Independent Elders Survey (MoVIES)	Participants 65 years of age and over, with 6^th ^grade or greater education, and living in the community were selected from voter registration lists in 23 communities in southwestern Pennsylvania	1147	Clinical assessments and in-person interviews in respondents' homes annually	1987–2001	156	Sociodemographics, functional and cognitive impairment, medication usage, depression

NOTE: ADL = activities of daily living; IADL = instrumental activities of daily living

### Predictors of nursing home admission

**Figure 2:**
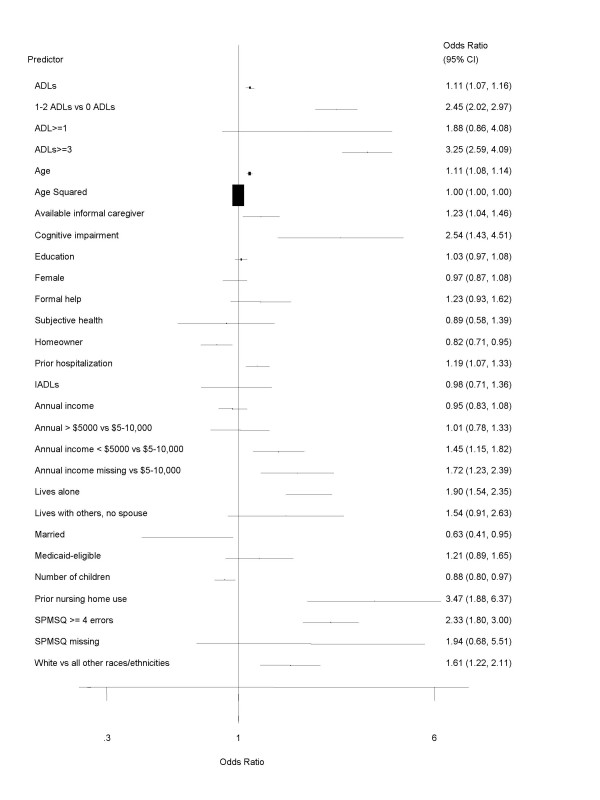
Predictors of nursing home admission: Logistic regression results (NOTE: ADL = activities of daily living; IADL = instrumental activities of daily living).

Table 2 presents the results of the random effects models for studies of NH entry that relied on logistic regression analyses. Several sociodemographic variables emerged as significant predictors of NH admission across the various data sources. Greater age, an annual income of less than $5,000 or a missing report of income (when compared to a reference of $5,000–$10,000 annual income; in 1982 dollars), and Caucasian race/ethnicity (when compared to all other racial/ethnic types) were significantly associated with greater odds of subsequent NH entry. As presented in Figure 2, the strength of these relationships varied, with the missing income and Caucasian variables appearing to exert the strongest influence on NH admission when compared to the other sociodemographic indicators.

**Table 2:** Summary Odds Ratios for Predictors of Nursing Home Admission in Community-Dwelling Older Adults

**Predictor**	**Data Sources Included (% of Participants Who Entered Nursing Home)**	**Pooled Odds Ratio (95% Confidence Interval)**	***P *Value for Heterogeneity**
ADLs	LSOA(14.6) [10], EPESE-IOWA(11.9) [34], NLTCS (14.8) [24]	1.11 (1.07–1.16)	.73
1–2 ADLs (versus 0 ADLs)	EPESE-EB(4.0) [21], EPESE-NH(9.0) [21], EPESE-IOWA(12.0) [21], NLTCS (14.8) [15], LSOA(n/a) [11]	2.45 (2.02–2.97)	.87
ADLs >= 1	MHCPS(9.1) [33], GAO(25.4) [35]	1.88 (.86–4.08)	.01
ADLs >= 3	EPESE-EB(4.0) [21], EPESE-NH(9.0) [21], EPESE-IOWA(12.0) [21], NLTCS(14.8) [15], LSOA(n/a) [11]	3.25 (2.59–4.09)	.30
Age	MHCPS(9.1) [36], LSOA(14.6) [10], EPESE-EB(4.0) [21], EPESE-NH(9.0) [21], EPESE-IOWA(12.0) [21], MEHCP(24.4) [26], NLTCS(8.0) [23], AHEAD(9.7) [28], GAO(25.4) [35]	1.11 (1.08–1.14)	.00
Age squared	MS(2.9) [32], EPESE-IOWA(11.9) [34], AHEAD [28]	1.00 (1.00–1.01)	.00
Available informal caregiver	LSOA(14.6) [10], NLTCS(8.0) [23], GAO(25.4) [35]	1.23 (1.04–1.46)	.57
Cognitive impairment	NLTCS(16.9) [13], MEHCP(24.4) [26], MHCPS(9.1) [37]	2.54 (1.44–4.51)	.04
Education	MHCPS(9.1) [36], EPESE-IOWA(11.9) [34], GAO(25.4) [35]	1.03 (.97–1.08)	.20
Female	MHCPS(9.1) [36], LSOA(14.6) [10], EPESE-EB(4.0) [21], EPESE-NH(9.0) [21], EPESE-IOWA(12.0) [21], NLTCS(14.8) [15], AHEAD(9.7) [28], GAO(25.4) [35]	.97 (.87–1.08)	.02
Formal help	LSOA(14.6) [10], NLTCS(14.8) [15]	1.23 (.93–1.62)	.47
Subjective health	MHCPS(9.1) [36], EPESE-IOWA(11.9) [34], LSOA(3.9) [27]	.90 (.58–1.39)	.00
Homeowner	LSOA(14.6) [10], NLTCS(16.9) [13]	.82 (.71–.95)	.21
Prior hospitalization	LSOA(14.6) [10], NLTCS(16.9) [13], EPESE-IOWA(11.9) [34]	1.19 (1.07–1.33)	.67
IADLs	LSOA(14.6) [10], NLTCS(8.0) [23]	.98 (.71–1.36)	.00
Income	NLTCS [11], EPESE-IOWA(11.9) [34], AHEAD(9.7) [28], GAO(25.4) [35]	.95 (.83–1.08)	.00
Annual income > $5,000 (vs. $5,000–$10,000)	EPESE-EB(4.0) [21], EPESE-NH(9.0) [21], EPESE-IOWA(12.0) [21]	1.02 (.78–1.33)	.82
Annual income < $5,000 (vs. $5,000–$10,000)	EPESE-EB(4.0) [21], EPESE-NH(9.0) [21], EPESE-IOWA(12.0) [21]	1.45 (1.15–1.83)	.70
Annual income missing (vs. $5,000–$10,000)	EPESE-EB(4.0) [21], EPESE-NH(9.0) [21], EPESE-IOWA(12.0) [21]	1.72 (1.23–2.39)	.16
Lives alone	MHCPS(9.1) [36], EPESE-EB [21], EPESE-NH [21], EPESE-IOWA [21], NLTCS(15.7) [14], LSOA(3.9) [27], GAO(25.4) [35]	1.91 (1.55–2.35)	.02
Lives with others, no spouse	EPESE-EB(4.0) [21], EPESE-NH(9.0) [21], EPESE-IOWA(12.0) [21]	1.54 (.91–2.63)	.12
Married	LSOA(14.6) [10], EPESE-IOWA(11.9) [34], AHEAD(9.7) [28]	.63 (.41–.95)	.01
Medicaid-eligible	MHCPS(9.1) [36], MS(2.9) [32], LSOA(14.6) [10], NLTCS(14.8) [15]	1.21 (.89–1.65)	.00
Number of children	LSOA(14.6) [10], NLTCS(14.8) [24]	.88 (.80–.97)	.00
Prior nursing home use	EPESE-EB(4.0) [21], EPESE-NH(9.0) [21], EPESE-IOWA(12.0) [21], MHCPS(2.9) [33], NLTCS(14.8) [15]	3.47 (1.89–6.37)	.00
SPMSQ >= 4 errors	EPESE-EB(4.0) [21], EPESE-NH(9.0) [21], EPESE-IOWA(12.0) [21]	2.33 (1.80–3.00)	.48
SPMSQ missing	EPESE-EB(4.0) [21], EPESE-NH(9.0) [21], EPESE-IOWA(12.0) [21]	1.94 (.68–5.51)	.01
White (vs. all other races/ethnicities)	LSOA(14.6) [10], NLTCS(15.7) [14], AHEAD(9.7) [28]	1.61 (1.22–2.11)	.04

NOTE: ADL = activities of daily living; IADL = instrumental activities of daily living

Several variables representing potential sources of support (formal or informal) also appeared to increase the risk of NH admission. Older adults who were married or had more living children had lower odds to enter NHs, whereas older adults who lived alone had nearly twice the odds to be admitted. Older adults with an available caregiver had greater odds to enter a NH, which is in contrast to some conclusions of the importance of informal care [5-7]. Those who had been hospitalized prior to interview had slightly greater odds to experience NH entry, whereas prior NH use was a strong predictor of subsequent admission (see Figure 2).

Indicators of functional and cognitive impairment were among the strongest predictors of NH admission. Older adults who indicated 3 or more ADL dependencies had considerably greater odds to enter NHs. The presence of cognitive impairment, assessed through either proxy/other subjective measures or 4 or more errors on clinically validated short screening tools such as the Short Portable Mental Status Questionnaire [40] were strongly linked to subsequent NH admission (see Figure 2).

**Figure 3:**
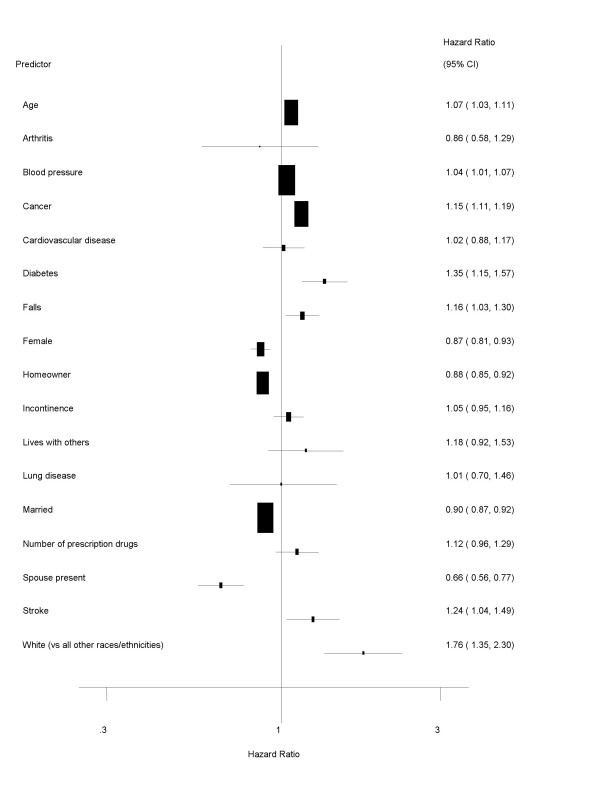
Predictors of nursing home admission: Cox regression results (NOTE: ADL = activities of daily living; IADL = instrumental activities of daily living).

Increased age and Caucasian race/ethnicity were predictive of earlier NH entry in the Cox regression results whereas being married and owning a home were associated with delayed admission (see Table 3 and Figure 3). Unlike the logistic regression model, female gender was significantly predictive of NH admission as women appeared less likely to enter NHs than men. The Cox regression results also reported on several additional predictors of NH admission; four health conditions emerged as triggering expedited NH admission (presence of diabetes, high blood pressure, cancer, and stroke). Falls also appeared as a significant, albeit rather moderate, predictor of earlier NH admission. Older adults with a spouse present or who were married also entered NHs later.

**Table 3:** Summary Hazard Ratios for Predictors of Time to Nursing Home Admission in Community-Dwelling Older Adults

**Predictor**	**Data Sources Included (% of Participants Who Entered Nursing Home)**	**Pooled Hazards Ratio (95% Confidence Interval)**	*P *Value for Heterogeneity*
Age	LSOA(14.6^a^) [12], MHOS(9.2) [31], MoVIES(13.6) [38], NLTCS(7.6^b^) [22]	1.07 (1.03–1.11)	.00
Arthritis	AHEAD(17.0) [29], MHOS(9.2) [31]	.86 (.58–1.29)	.00
Blood pressure	AHEAD(17.0) [29], MHOS(9.2) [31]	1.04 (1.01–1.07)	.60
Cancer	AHEAD(17.0) [29], MHOS(9.2) [31]	1.15 (1.11–1.19)	.68
Cardiovascular disease	AHEAD(17.0) [29], MHOS(9.2) [31]	1.02 (.88–1.17)	.32
Diabetes	AHEAD(17.0) [29], MHOS(9.2) [31]	1.35 (1.15–1.57)	.11
Falls	AHEAD(17.0) [29], MHOS(9.2) [31]	1.16 (1.02–1.30)	.28
Female	AHEAD(17.0) [29], EPESE-NH(33.3) [39], LSOA(14.6) [12], MHOS(8.2) [31], MoVIES(13.6) [38], NLTCS(7.6) [22]	.87 (.81–.93)	.18
Homeowner	LSOA(14.6) [12], MHOS(8.2) [31]	.88 (.85–.92)	.61
Incontinence	AHEAD(17.0) [29], EPESE-NH(33.3) [39]	1.05 (.95–1.17)	.34
Living with others	LSOA(14.6) [12], MoVIES(13.6) [38]	1.23 (.93–1.62)	.89
Lung disease	AHEAD(17.0) [29], MHOS(8.2) [31], NLTCS(7.6) [22]	1.01 (.70–1.46)	.00
Married	MHOS(8.2) [31], MoVIES(13.6) [38]	.90 (.87–.92)	.46
Number prescription drugs	EPESE-NH(33.3) [39], MoVIES(13.6) [38]	1.12 (.96–1.29)	.002
Spouse present	AHEAD(17.0) [29], EPESE-NH(33.3) [39]	.66 (.56–.77)	.24
Stroke	AHEAD(17.0) [29], EPESE-NH(33.3) [39]	1.24 (1.04–1.49)	.05
White (vs. all other races/ethnicities)	EPESE-NH(33.3) [39], MHOS(8.2) [31], NLTCS(14.0) [25]	1.76 (1.35–2.30)	.00

NOTE: ^a^Admission rate taken from LSOA data reported in Coward et al.^10^

^b^"Long-stay" admission rate from 1982–1984 in NLTC.

### Publication bias and heterogeneity

Due to the relatively small numbers of studies within each reported predictor of NH admission, we did not formally assess publication bias. The tests of heterogeneity for each predictor, as shown in Tables 3 and 4, suggest that for several predictors statistical heterogeneity was present. In some cases heterogeneity was present for significant predictors of entry (age, cognitive impairment, lives alone, married, number of children, Caucasian race) and time to NH admission (age, Caucasian race). Random effects models were chosen for the meta-analysis *a priori *in part to address these concerns. Stratification, subgroup analyses, and meta-regression approaches were considered [19], but the number of data sources available for each predictor precluded a more detailed assessment of heterogeneity. While some methodologists consider the pooling of empirical associations from heterogeneous results as invalid, others suggest that a pooled estimate of heterogeneous associations can provide an unbiased summary [41-43]. Moreover, visual inspection of effect sizes by the 1^st ^and 3^rd ^authors found minimum variation across data sources for each predictor, implying less clinically-significant heterogeneity than the statistical tests suggest [19,43].

## Discussion

Our results and subsequent interpretations are bolstered by the adherence to systematic review principles in meta-analysis [19,44]. This includes the reliance on separate data sources (as opposed to independent studies), systematic identification and retrieval of studies, a cross-referencing approach conducted to the point of saturation, and a reliance on studies and data sources that were representative of the elderly population in the U.S. or a region thereof. However, there were several limitations to this meta-analysis that are important to note. Few studies distinguished between short-term NH stays for rehabilitative purposes and long-term admissions [22]. Due to available resources, only the principal author screened available abstracts and extracted data for the meta-analysis. Guidelines to establish quality in meta-analyses emphasize that inter-rater agreement of multiple reviewers enhances the validity of the data extracted and synthesized in systematic reviews [19,44]. Multiple studies and their data sources either did not report complete data for the purposes of the meta-analysis or utilized analytical approaches that were unable to be integrated into the pooled findings reported in this study (e.g., multinomial logistic regression). Similarly, as it is an issue in all meta-analyses of observational studies, extracted predictors of NH admission were derived from studies that included models with varying numbers of adjusting covariates; variations in adjustment across predictive models pose challenges to standardization when pooling estimates in meta-analyses. Related to this issue, variation in the outcome rates (i.e., percentages of participants who entered NHs; see Tables 2 and 3) may have led to the observed statistical heterogeneity. Concerns have also been raised in the use of odds ratios as measures of association (the most common statistic used to report pooled effects in meta-analyses), particularly in instances where there is existence of heterogeneity [45].

Several of the results showed significant heterogeneity, suggesting a lack of sensitivity in the pooled empirical associations [19]. The published data available did not offer the opportunity to test the clinical sources of heterogeneity (i.e., subgroup or meta-regression analyses) beyond visual inspection of effect sizes across data sources and predictors. This is a potential weakness of meta-analyses, particularly when attempting to synthesize across observational studies (as opposed to randomized controlled trials), as various methods related to sampling, measurement, and research design could mask associations between predictors and clinical outcomes of interest.

Another important limitation to note is that the meta-analysis was limited to individual predictors of NH admission. Interactions between sociodemographic characteristics, indicators of functional impairment, and similar predictors is likely to occur given the wide range of variables that potentially influence NH entry. The reliance on published data limited this meta-analysis to pool similar variables across studies; future meta-analysis of predictors of NH admission or other healthcare transitions could utilized pooled individual data methods to conduct a meta-analysis of key interaction terms [20,43].

In order to maximize the relevance of these findings, we relied on the pooled logistic regression estimates to determine whether an older patient is at risk for NH admission or not at some point in the future (i.e., 1 year or more). Older adults with 3 or more ADL dependencies had approximately 3.25 times the odds to enter a nursing over a 2–6 year interval. Similarly, elderly patients with 4 or more errors on a short screening tool (Short Portable Mental Status Questionnaire) [40] had more than twice the odds to enter a NH 3 years in the future. In some instances, older adults or their caregivers have to deal with the consequences of a cataclysmic event (e.g., an injurious fall) [46] that sets into motion a cascade of crises where instant and unavoidable long-term care decisions are made on an ad-hoc basis. However, awareness of the important thresholds reported here may inform older adults and their caregiving families in the years prior to a potential admission event. Earlier intervention in the long-term care decision-making process may also prompt the mobilization of community-based resources or clinical services to forestall a NH admission [47].

The findings in this meta-analysis suggest that once certain functional or cognitive thresholds are reached, future risk of NH admission increases substantially (net a host of other factors). As suggested in prior research, intervention in the earlier stages of a chronic disease trajectory that offers respite and support to older adults or their caregiving families may help to potentially delay NH entry [47,48]. However, experimental research (such as randomized controlled trials) would better inform whether intervention in earlier stages of chronic disease trajectories for older adults may help to delay clinical outcomes such as NH admission.

The results may offer useful prognostic information for clinicians, families, and older patients. For example, the significant predictors from the current meta-analyses could be converted into a practical screening tool of NH admission risk. A series of single dichotomous codes (1 = yes; 0 = no) could be applied for each indicator and then further weighted according to the effect size reported here. Summing these numbers and standardizing the sum would create a "risk score" on a 0–10 metric that offers guidance as to whether a geriatric patient is at risk for NH entry in the future. An almost identical algorithm has been utilized to predict NH admission in a smaller, clinic-based sample of persons with Alzheimer's disease where presence of a particular risk factor was calculated (i.e., the "value;" 1 = present; 0 = absent) and multiplied with the coefficient value of that risk factor as a weight (derived from Cox regression models) [16]. The products of the values and weights were then summed, and this value was successfully applied as a prognostic tool to predict NH care and death among a second, validation sample of individuals with Alzheimer's disease. Relying on published data for this meta-analysis did not allow us to test the predictive accuracy of this proposed prognostic index because individual level data were not available to model. The next step in testing such a tool is to apply it to individual patient data [20,43] across nationally representative samples (e.g., the publicly available data sources described in Table 1) and determine its specificity and sensitivity to NH admission over various follow-up intervals, thus offering empirical evidence for its efficacy as a screening tool for risk of NH entry. It is important to note that similar tools used to predict clinical outcomes such as hospital admission have explained areas under receiver operant curves of .70 or below [49]. This suggests that creating risk prognostic tools are helpful for population- or group-based planning strategies but less advantageous for individual clinical decision-making.

Approaching the prediction of future NH admission in terms of a threshold model may provide improved accounting of risk. Studies that have incorporated cut-points on functional or cognitive variables have generally accounted for greater variance in NH entry (Cox-Snell R^2 ^of .40–.42) [21] and the results of this meta-analysis confirm such an approach when identifying the presence or absence of risk factors for inclusion in predictive algorithms. However, a considerable amount of unexplained variance in the prediction of NH admission still remains in many studies. Predictions across several years are always risky because too many intervening events may occur. Nonetheless, adapting more complex conceptual models that differentiate between long-term predictors and immediate crisis events may illuminate the process leading to immediate or future NH entry, and may inform clinicians of the need to direct patients to early community-based service use (when distal predictors such as those presented here are identified) or develop immediate care plans for those at impending risk of NH admission.

Future descriptive and clinical research could approach NH placement as less of an endpoint and instead as an important transition where timing of NH admission, type and characteristics of the institution, and preplacement factors all operate to influence outcomes after admission. Relatively little research has explored these issues or has considered data at these variable levels of analysis when examining the impact of the NH transition on key health outcomes [4,50]. By building research in this area, future clinical efforts could not only strive to delay placement, but also improve patient and family outcomes if such an event must occur.

## Conclusion

Relying on multiple and representative sources of data, we have identified a number of indicators that predict NH admission over multi-year intervals. A lack of informal support or socioeconomic resources appeared to strongly precipitate entry. The most powerful predictors were those functional variables with identified cut-points, such as ADLs or cognitive impairment. The findings suggest a threshold effect in certain functional or cognitive dimensions that may signal the initiation of the admission process. As expected, the results here confirm those reported in prior well-designed, systematic reviews, where ADL dependencies, cognitive impairment, non-Caucasian race/ethnicity, prior NH admission, and social support/caregiving factors are identified as important precursors of entry [5-7]. However, the findings reported in this meta-analysis extend these original insights by providing pooled effect estimates that specify the empirical strength of relationships between potential predictors, their cut-points, and NH admission. Systematic reviews cannot offer such specificity, as they are limited to summarizing or counting the significant relationships among included studies [19]. Not only do the pooled associations provide detailed empirical information as to which variables emerge as the strongest predictors of NH admission (e.g., 3 or more ADL dependencies, cognitive impairment, prior NH use), but these reported associations could also be utilized as weights in the construction and validation of prognostic tools to estimate risk for NH entry over a multi-year period. The results extend the findings of past review efforts in two other key ways: 1) the reliance on representative data sources yielded empirical estimates that are more generalizable than prior efforts that integrate findings from representative and non-representative individual studies; and 2) each data source was treated as an observation in the meta-analysis, which avoids treating results from multiple studies on the same data source as individual observations. In this regard, the current findings build on the impressive efforts of prior reviews to provide empirical estimates of NH admission.

## Competing interests

The author(s) declare that they have no competing interests.

## Authors' contributions

JEG conducted the database search, screened and extracted data for the meta-analysis, prepared extracted data for the meta-analysis procedures, and had primary responsibility in writing this article.

SD conducted the meta-analyses, provided JEG with visual illustrations of the pooled results, and provided critiques and guidance in the Methods and Results section of the article. She also participated in the writing of this paper.

KAA obtained the majority of studies used in the meta-analysis, assisted JEG in managing and filing these studies for the purposes of the meta-analysis, and also reviewed the article for editing purposes.

RLK provided conceptual guidance throughout the meta-analysis, assisted JEG in the review of extracted data, edited sections throughout the manuscript (particularly the Introduction and Discussion), and aided JEG in interpreting the clinical significance of the results. He also participated in the writing of this paper.

## Pre-publication history

The pre-publication history for this paper can be accessed here:



## References

[B1] The American Geriatrics Society Foundation for Health in Aging. http://www.healthinaging.org/agingintheknow/chapters_print_ch_trial.asp?ch=15.

[B2] Wunderlich GS, Kohler P, (Eds) (2001). Improving the Quality of Long-Term Care (Report of the Institute of Medicine).

[B3] Kane RA (2001). Long-term care and a good quality of life: bringing them closer together. Gerontologist.

[B4] Schulz R, Belle SH, Czaja SJ, McGinnis KA, Stevens A, Zhang S (2003). Long-term care placement of dementia patients and caregiver health and well-being. JAMA.

[B5] Miller EA, Weissert WG (2000). Predicting elderly people's risk for nursing home placement, hospitalization, functional impairment, and mortality: a synthesis. Med Care Res Rev.

[B6] Wingard DL, Jones DW, Kaplan RM (1987). Institutional care utilization by the elderly: a critical review. Gerontologist.

[B7] Kane RA, Kane RL (1987). Long-term care:Principles, programs, and policies.

[B8] Doty P (1986). Family care of the elderly: the role of public policy. Milbank Q.

[B9] Nygaard HA, Alberktsen G (1992). Risk factors for admission to a nursing home: a study of elderly people receiving home nursing. Scan J Prim Health Care.

[B10] Coward RT, Netzer JK, Mullens RA (1996). Residential differences in the incidence of nursing home admissions across a six-year period. J Gerontol Psychol Sci Soc Sci.

[B11] Steinbach U (1992). Social networks, institutionalization, and mortality among elderly people in the United States. J Gerontol.

[B12] Kersting RC (2001). Impact of social support, diversity, and poverty on nursing home utilization in a nationally representative sample of older Americans. Soc Work Health Care.

[B13] Hanley RJ, Alexcih LMB, Wiener JM, Kennell DL (1990). Predicting elderly nursing home admissions: results from the 1982–1984 National Long-Term Care Survey. Res Aging.

[B14] Kasper JD, Shore AD (1994). Cognitive impairment and problems behaviors as risk factors and institutionalization. J Appl Gerontol.

[B15] Pearlman DN, Crown WH (1992). Alternative sources of social support and their impacts on institutional risk. Gerontologist.

[B16] Stern Y, Tang MX, Albert MS, Jacobs DM, Bell K, Marder K, Sano M, Devanand D, Albert SM, Bylsma F, Tsai WY (1997). Predicting time to nursing home care and death in individuals with Alzheimer's disease. JAMA.

[B17] Gaugler JE, Edwards AB, Femia EE, Zarit SH, Stephens MAP, Townsend A, Greene R (2000). Predictors of institutionalization of cognitively impaired older adults: family help and the timing of placement. J Gerontol Psychol Sci Soc Sci.

[B18] Gaugler JE, Kane RL, Kane RA, Clay T, Newcomer R (2003). Predicting institutionalization of cognitively impaired older people: utilizing dynamic predictors of change. Gerontologist.

[B19] Petitti DB (2000). Meta-analysis, decision analysis, and cost-effectiveness analysis:Methods for quantitative synthesis in medicine.

[B20] Stewart LA, Tierney JF (2002). To IPD or not to IPD? Advantages and disadvantages of systematic reviews using individual patient data. Eval Health Prof.

[B21] Foley DJ, Ostfield AM, Branch LG, Wallace RB, McGloin J, Coroni-Huntley JC (1992). The risk of nursing home admission in three communities. J Aging Health.

[B22] Liu K, McBridge T, Coughlin T (1994). Risk for entering nursing homes for long versus short stays. Med Care.

[B23] Newman S, Struyk R, Wright P, Rice M (1990). Overwhelming odds: Caregiving and the risk of institutionalization. J Gerontol Psychol Sci Soc Sci.

[B24] Cutler DM, Sheiner LM, Wise DA (1994). Policy options for long-term care. Studies in the Economics of Aging.

[B25] Headen AEJ (1993). Economic disability and health determinants of the hazard of nursing home entry. J Hum Resour.

[B26] Jette AM, Tennstedt S, Crawford S (1995). How does formal and informal community care affect nursing home use?. J Gerontol Psychol Sci Soc Sci.

[B27] Speare A, Avery R, Lawton L (1991). Disability, residential mobility, and changes in living arrangements. J Gerontol Psychol Sci Soc Sci.

[B28] Himes CL, Wagner GG, Wolf DA, Aykan H, Dougherty DD (2000). Nursing home entry in Germany and the United States. J Cross Cult Gerontol.

[B29] Banaszak-Holl J, Fendrick AM, Foster NL, Herzog AR, Kabeto MU, Kent DM, Straus WL, Langa KM (2004). Predicting nursing home admission: estimates from a 7-year follow-up of a nationally representative sample of older Americans. Alzheimer's Disease and Associated Disorders.

[B30] Van-Houtven CH, Norton EC (2004). Informal care and health care use of older adults. J Health Econ.

[B31] Harris Y, Cooper JH (2006). Depressive symptoms in older people predict nursing home admission. Journal of the American Geriatrics Society.

[B32] Cohen M, Tell EJ, Wallack SS (1986). Client-related risk factors of nursing home entry among elderly adults. J Gerontol.

[B33] Jette AM, Branch LG, Sleeper LA, Feldman H, Sullivan LM (1992). High-risk profiles for nursing home admission. Gerontologist.

[B34] Russell DW, Cutrona CE, de la Mora A, Wallace RB (1997). Loneliness and nursing home admission among rural older adults. Psychol Aging.

[B35] Roy AW, Folmar S, Ford AB (1990). The elderly and risk factors for institutionalization: evidence from the Cleveland GAO Study, 1975–1984. J Appl Soc Sci.

[B36] Branch LG, Jette AM (1982). A prospective study of long-term care institutionalization among the aged. Am J Public Health.

[B37] Jette AM, Branch LG (1983). Targeting community services to high-risk elders: Toward preventing long-term care institutionalization. PrevHum Serv.

[B38] Bharucha AJ, Pandav R, Shen C, Dodge HH, Ganguli M (2004). Predictors of nursing facility admission: a 12-year epidemiological study in the United States. J Am Geriatr Soc.

[B39] Lachs MS, Williams CS, O'Brien S, Pillemer KA (2002). Adult protective service use and nursing home placement. Gerontologist.

[B40] Pfeiffer E (1975). A short portable mental status questionnaire for the assessment of organic brain deficit in elderly patients. J Am Geriatr Soc.

[B41] Peto R (1987). Why do we need systematic overviews of randomized trials?. Stat Med.

[B42] Fleiss JL, Gross AJ (1991). Meta-analysis in epidemiology, with special reference to studies of the association between exposure to environmental tobacco smoke and lung cancer: a critique. J Clin Epidemiol.

[B43] Friedenreich CM (1993). Methods for pooled analysis of epidemiologic studies. Epidemiology.

[B44] Stroup DF, Berline JA, Morton SC, Olkin I, Williamson GD, Rennie D, Moher D, Becker BJ, Sipe TA, Thacker SB, for the Meta-analysis Of Observational Studies in Epidemiology (MOOSE) Group (2000). Meta-analysis of observational studies in epidemiology: a proposal for reporting. JAMA.

[B45] Pepe MS, Janes H, Longton G, Leisenring W, Newcomb P (2004). Limitations of the odds ratio in gauging the performance of a diagnostic, prognostic, or screening marker. Am J Epidemiol.

[B46] Tinetti ME, Williams CS (1997). Falls, injuries due to falls, and the risk of admission to a nursing home. New Engl J Med.

[B47] Gaugler JE, Kane RL, Kane RA, Newcomer R (2005). Early community-based service utilization and its effects on institutionalization in dementia caregiving. Gerontologist.

[B48] Gaugler JE, Kane RL, Kane RA, Newcomer R (2005). The effects of duration of caregiving on institutionalization. Gerontologist.

[B49] Wagner JT, Bachmann LM, Boult C, Harari D, von Renteln-Kruse W, Egger M, Beck JC, Stuck AE (2006). Predicting the risk of hospital admission in older persons-validation of a brief self-administered questionnaire in three European countries. J Am Geriatr Soc.

[B50] Gaugler JE, Leitsch SA, Zarit SH, Pearlin LI (2000). Caregiver involvement following institutionalization: effects of preplacement stress. Res Aging.

